# Supplementary material for: Spatial Immunology in Liver Metastases from Colorectal Carcinoma according to the Histologic Growth Pattern
Source: Cancers (Basel). 2022 Jan 29;14(3):689. doi: 10.3390/cancers14030689 (PMC8833601; doi:10.3390/cancers14030689)
Supplement: Supplementary file 1 [file cancers-14-00689-s001.zip › cancers-1546246-supplementary.pdf]

cancers

Supplementary material and methods

The following combinations of retrieval buffers, primary antibodies, amplification systems and Opal fluorophores were used:

- CD4 (Mouse/4B12, DAKO/M7310, 1:100) - ImmPRESS™- HRP Horse Anti-Mouse IgG Polymer Detection Kit, Peroxidase MP-7500, Vector Laboratories, Burlingame, CA- Opal 520
- CD8α (Mouse/144B, Thermo Fisher Scientific/MA5-13473, 1:200) - ImmPRESS™- HRP Horse Anti-Mouse IgG Polymer Detection Kit, Peroxidase MP-7500, Vector Laboratories, Burlingame, CA- Opal 570
- CD20 (Mouse/L26, DAKO/GA604, 1:1500) - ImmPRESS™- Opal Polymer HRP Ms+Rb, Akoya - Opal 540
- FoxP3 (Rabbit/ D6O8R, Cell Signaling Technology/12653s, 1:100) - ImmPRESS™- HRP Horse Anti-Rabbit IgG Polymer Detection Kit, Peroxidase MP-7401, Vector Laboratories, Burlingame, CA- Opal 620
- CD45RO (Mouse/UCHL1, Thermo Fisher Scientific/MA1-19452, 1:100) - ImmPRESS™- Opal Polymer HRP Ms+Rb, Akoya - Opal 650
- Pan-cytokeratin cocktail [anti-E-cadherin (Mouse/Clone 36, BD Biosciences/610182, 1:2000), anti-pan Cytokeratin (Mouse/[C-11], Abcam, San Francisco/ ab7753, 1:500) and anti-pan Cytokeratin Clone, 1:200 (Mouse/AE1/AE3, Thermo Fisher Scientific/MA5-13156, 1:500)] - ImmPRESS™- HRP Horse Anti-Mouse IgG Polymer Detection Kit, Peroxidase MP-7500, Vector Laboratories, Burlingame, CA- Opal 480
- ASMA (clone 1A4, M0851, DAKO, 1:100) - ImmPRESS™- HRP Horse Anti-Mouse IgG Polymer Detection Kit, Peroxidase MP-7500, Vector Laboratories, Burlingame, CA- Opal 780.

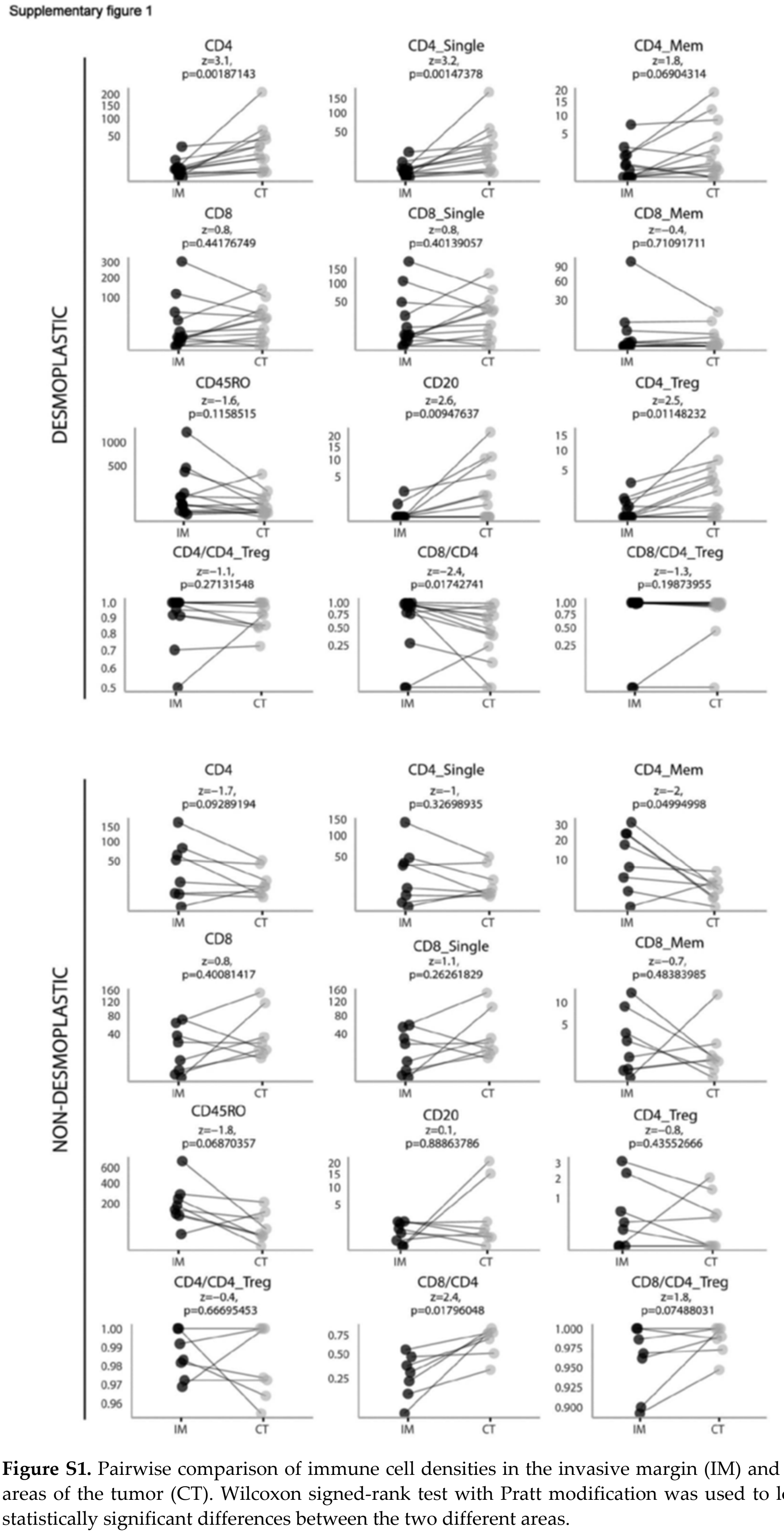

Figure S1. Pairwise comparison of immune cell densities in the invasive margin (IM) and central areas of the tumor (CT). Wilcoxon signed-rank test with Pratt modification was used to look for statistically significant differences between the two different areas.
